# MiR-16-5p mediates a positive feedback loop in EV71-induced apoptosis and suppresses virus replication

**DOI:** 10.1038/s41598-017-16616-7

**Published:** 2017-11-27

**Authors:** Caishang Zheng, Zhenhua Zheng, Jianhong Sun, Yuan Zhang, Chunyu Wei, Xianliang Ke, Yan Liu, Li Deng, Hanzhong Wang

**Affiliations:** 1 Guangzhou Institute of Pediatrics, Guangzhou Women and Children Medical Center, Guangzhou, 510623 China; 2 CAS Key Laboratory of Special Pathogens and Biosafety, Wuhan Institute of Virology, Chinese Academy of Sciences, Wuhan, 430071 China

## Abstract

Enterovirus 71 (EV71) is the predominant causative pathogen of hand-foot-and-mouth disease (HFMD). Contrary to other HFMD-causing enterovirus, EV71 can lead to severe neurological complications, even death. MicroRNAs (miRNAs) are small non-coding RNAs that constitute the largest family of gene regulators participating in numerous biological or pathological processes. We previously reported that miR-16-5p increases with severity of HFMD by investigating the expression patterns of host miRNAs in patients with HFMD. However, the mechanisms by which EV71 induces miR-16-5p expression are not clear, and the interaction between EV71 and miR-16-5p is not yet fully understood. Here, we confirmed EV71-induced expression of miR-16-5p both *in vitro* and *in vivo* and show that upregulation of miR-16-5p by EV71 infection may occur at the posttranscriptional level. Moreover, EV71-induced caspase activation facilitates the processing of pri-miR-16-1. We also revealed that miR-16-5p can promote EV71-induced nerve cells apoptosis through activating caspase-3. In addition, we found that miR-16-5p can inhibit EV71 replication. CCNE1 and CCND1, two important cell cycle regulators, play an important role in the suppression of EV71 replication by miR-16-5p. Therefore, miR-16-5p is a positive feedback regulator in EV71-induced apoptosis and a suppressor of virus replication. These results help in understanding the interaction network between miRNA and EV71 infection and provide a potential target for the development of antiviral therapy.

## Introduction

Enterovirus 71 (EV71) is a single-positive-stranded RNA virus belonging to the *Enterovirus* genus of the Picornaviridae family^1,2^. EV71 is identified as one of the causative pathogens of infant hand-foot-and-mouth disease (HFMD) and transmitted through faecal-oral and respiratory routes^3^. Although HFMD is generally asymptomatic or presents benign symptoms, the disease may also lead to severe neurological complications^4,5^. Similar to poliovirus, acute EV71 infection can cause severe neurological complications, including myocarditis, aseptic meningitis, encephalitis, acute flaccid paralysis, pulmonary oedema or haemorrhage and even fatality^1,6–8^. Children under five years old are the mainly susceptible to severe EV71 infection^9^. Since being first reported in 1974, EV71 has induced several epidemic outbreaks in the world, particularly in the Asia-Pacific region^10–12^. However, specific antiviral therapies for the treatment of HFMD patients are currently unavailable because of high genomic mutation rate and lack of understanding on EV71 pathogenesis^13^. The underlying mechanisms through which EV71 infection induces serious cerebral and pulmonary complications and even death are unclear^14^. Therefore, further investigation on the pathogenesis of EV71 infection has kindled considerable research interest in the field of medicine and biology.

MicroRNAs (miRNAs) are approximately 19–24 nt non-coding RNAs that post-transcriptionally repress gene expression by targeting messenger RNAs (mRNAs)^15,16^. MiRNAs were firstly identified in *Caenorhabditis elegans* and are widely expressed in vertebrates, plants and several DNA viruses^15,17^. To date, >5500 miRNAs have been predicted throughout the human genome with numerous limited to specific tissues^18,19^. A total of 28645 miRNA have been annotated in the current version of the miRNA database (the miRBase Sequence Database–Release 21). Given that a single miRNA may bind up to 100 different transcripts, these miRNAs regulate the expression a large number of genes participating in multiple cellular processes, such as development, differentiation, growth, homeostasis, stress responses, apoptosis and host-pathogen interactions^20–22^. Most miRNA genes are embedded in either independent noncoding RNAs or the introns of protein-coding genes and transcribed for the most part by RNA polymerase II as long primary transcripts (pri-miRNA), which are characterised by hairpin structures^23–25^. Then, pri-miRNA is recognised and processed into pre-miRNA by the microprocessor complex, which consists of the RNAse III enzyme, DROSHA and co-factor DiGeorge syndrome critical region 8 (DGCR8)^26–28^. The liberated pre-miRNA is exported into the cytoplasm by Exprotin 5 (XPO5) and RanGTP^29,30^. In the cytoplasm, pre-miRNA is further cleaved by the RNAse III enzyme, DICER, as guided by the RNA-binding protein (TRBP), producing ~22 bp miRNA duplex intermediates bearing 2 nt 3′ overhangs at each end^31,32^. One strand of the duplex interacts with the RNA-induced silencing complex (RISC) and guides the RISC to target genes through complementary binding of the seed sequences; meanwhile, the other strand is degraded^33,34^. Mature miRNAs typically bind to complementary sequences, which are mainly found in the 3′ untranslated regions of target mRNAs and can inhibit translation and/or decrease mRNA stability^16,20^.

MiRNAs play a pivotal role in the complicated interaction networks between virus and host^35–38^. In general, viruses have evolved numerous strategies to overcome environmental stresses and host immune reactions to increase competitive advantages^17,39,40^. On one hand, numerous cellular miRNAs could directly bind to RNA virus genome to affect virus replication. For example, miR-122 can bind to HCV genomic RNA and increase viral RNA stability and viral replication^17,41,42^; miR-296-5p and miR-23b can bind to EV71 RNA and inhibit viral proteins translation^43,44^. On the other hand, viruses could modulate the expression of host miRNA levels during viral infections possibly because of both host antiviral defences and viral factors altering the cellular environment. For example, miR-146a is upregulated in several virus infections, such as DNEV, JEV and EV71, and inhibits the expression of interferon α/β expression by targeting TRAF6, a key molecule in the TLR signalling pathway^45–47^. To date, novel interactions between virus and host miRNA have rapidly been discovered with the development of deep sequencing and microarrays technologies^48,49^. However, in most cases, the biological significance and the underlying mechanisms of these virus–host interactions have yet to be determined. Therefore, the study of the interaction of virus and host miRNA will provide molecular insights not only into viral infection but also host gene regulation mechanisms.

To date, specific antiviral therapies for EV71-induced severe HFMD-associated diseases are not available^1^. Given the high genomic mutation rate of EV71, an effective protection vaccine for different strains of EV71 is difficult to develop^13^. Thus, a comprehensive understanding of the interaction between EV71 and host miRNA is urgently needed. In our previous work, we attempt to determine the miRNA expression profiles in sera of heathy children and children with mild and severe HFMD. We found that miR-16-5p was upregulated in exosomes extracted from sera of children with HFMD relative to those of health children^50^. Interestingly, we found that the expression of miR-16-5p is associated with the severity of HFMD, with the expression being higher in the severe group compared with the mild group^50^. However, the function of miR-16-5p in EV71 infection has yet to be revealed.

In this study, we confirmed the upregulation of miR-16-5p by EV71 infection both *in vitro* and *in vivo*. We further found that the upregulation of miR-16-5p by EV71 may occur during the processing of pri-mir-16-1 and is associated with EV71-induced activation of caspase. Interestingly, we revealed that miR-16-5p could promote EV71-induced nerve cells apoptosis. Moreover, miR-16-5p was able to inhibit EV71 replication by the suppression of the expression levels of the cell cycle regulators, cyclin E1 (CCNE1) and cyclin D1 (CCND1). Taken together, the results show that miR-16-5p is a positive feedback regulator in EV71-induced apoptosis and suppresses virus replication by targeting CCND1 and CCNE1.

## Results

### MiR-16-5p is highly induced by EV71 infection

**Figure 1:**
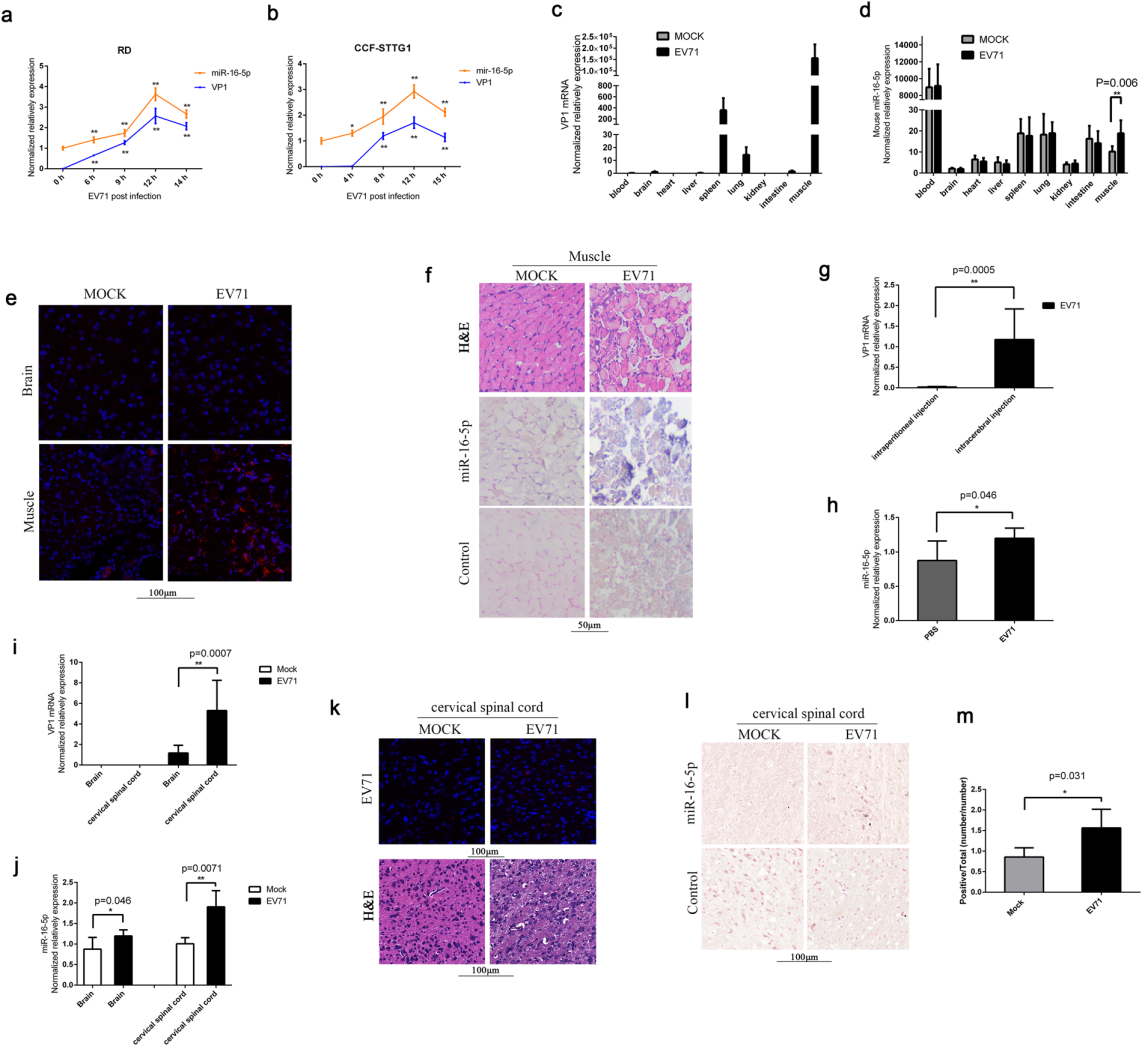
Expression levels of miR-16-5p in EV71-infected RD, CCF-STTG1 cells and EV71-infected mouse tissues. (**a**,**b**) The expression levels of hsa-miR-16-5p and EV71 VP1 gene were quantified by qRT-PCR after EV71 infection for 0, 6, 9, 12 and 14 (RD cells) or 0, 4, 8, 12 and 15 h (CCF-STTG1 cells). U6 rRNA was used as an internal control for the expression miR-16-5p. GAPDH was measured as a control for the expression of EV71 VP1gene. Values are means from triplicate experiments and represent the relative levels of expression in RD (**a**) and CCF-STTG1 (**b**) cells. The differences between 0 h and the other time points have been evaluated by statistical analysis respectively. (**c**,**d**) The expression of mmu-miR-16-5p was measured in EV71-infected mice. Two-week old KM mice (about 7–9 g) were intraperitoneally injected with 1 × 10^5^ TCID50 EV-A71 GZ-CII. After three to four days, the mice were sacrificed after appearing serious posterior paresis. The whole blood and different tissues from sacrificed mice were harvested for RNA extraction. The expression levels of the EV-A71 GZ-CII gene was quantified by qRT-PCR with specific primers for VP1 genes and with mouse GAPDH as an internal control (**c**). The expression of mmu-miR-16-5p was determined in different tissues (**d**). The relative expression levels of the target genes were calculated according to the 2^−∆∆*Ct*^ method. (**e**) Detection of EV71 replication in EV71-infected brain and muscle tissues by immunofluorescent histochemical staining with a specific anti-EV71 VP1 protein antibody (the red signals represent EV71 VP1 protein). (**f**) Hematoxylin and eosin staining of EV71-infected and mock-infected muscle tissue (upper panels).The expression levels of mmu-miR-16-5p in EV71-infected mock-infected muscle tissues were detected by *in situ* hybridisation (lower panels). (**g**) The expression levels of the EV-A71 GZ-CII gene in brain from the mice infected with virus through two different ways of injection were quantified by qRT-PCR. Two group of two-week old KM mice (about 7–9 g), one group were intraperitoneally injected with 1 × 10^5^ TCID50 EV-A71 GZ-CII and the other group were intracerebrally injected with 1 × 10^3^ TCID50 EV-A71 GZ-CII. After three to four days, the mice were sacrificed after appearing serious posterior analysis and the brains were separated for expression analysis. (h) Analysis the expression of mmu-miR-16-5p in brain from intracerebrally injected mice. Two group of two-week old KM mice (about 7–9 g), one were intracerebrally injected with 1 × 10^3^ TCID50 EV-A71 GZ-CII and the other group were intracerebrally injected with equal volume of PBS (5 μL). The mice were sacrificed and the brains were separated for expression analysis. (**i,j**) Analysis the expression of EV-A71 GZ-CII and mmu-miR-16-5p in whole brain and in cervical spinal cord from EV-A71 GZ-CII infected mice. After intracerebral injection with EV-A71 GZ-CII, the mice were sacrificed. The whole brain and cervical spinal cord were separated for expression analysis. The expression of EV-A71 GZ-CII (**i**) and mmu-miR-16-5p (**j**) were quantified by qRT-PCR. (**k**) The cervical spinal cords were separated from intracerebrally infected mice and were blocked in paraffin. The expression of EV-A71 GZ-CII was analysed by fluorescence immunohistochemistry with a specific anti-EV71 VP1 protein antibody (the red signals represent EV71 VP1 protein) (upper panels). The cervical spinal cords form EV-A71 GZ-CII infected and mock infected mice were stained by Hematoxylin and eosin (lower panels). (**l**,**m**) The expression of mmu-miR-16-5p in cervical spinal cords form EV-A71 GZ-CII intracerebrally infected mice were analysis by *in situ* hybridisation (**l**). Image analyses were undertaken using Aperio ImageScope Software (v10) and the Positive Pixel Count V9 algorithm (default settings) (**m**). The data represent mean ± SD and were analyzed by Student’s *t* test. Asterisks denote significant differences between indicated samples (*p < 0.05, **p < 0.01).

In our previous study, we found that the expression level of miR-16-5p was significantly induced in exosomes from the sera of children with HFMD compared with those from healthy children. Moreover, the expression of miR-16-5p is correlated with HFMD severity^50^. The expression level of miR-16-5p was higher in severe HFMD, mainly because of EV71 with severe neurologic clinical symptoms, compared with mild HFMD, without severe neurologic clinical symptoms^50^. To confirm the expression change of miR-16-5p during EV71 infection, we examined the expression levels of miR-16-5p in human rhabdomyosarcoma (RD) cells and human astrocytoma (CCF-STTG1) cells at the indicated times after EV71 infection. The expression level of miR-16-5p was markedly induced both in RD and CCF-STTG1 cells with EV71 infection (Fig. 1a and b). Furthermore, we used an EV71-infected mouse model to measure the expression of miR-16-5p *in vivo*. Two-week old Kunming (KM) mice were intraperitoneally injected with high-virulence EV71 (GZ-CII strain), and the mice were sacrificed after three or four days after appearing serious posterior paralysis. Through quantitative polymerase chain reaction (qPCR) assay, we found that the virus mostly exists in muscle (Fig. 1c). As shown in Fig. 1c, we also detected relatively low EV71 virus in brain, spleen, lung and intestine. We further assayed the expression levels of EV71 VP1 protein in brain and muscle by immunofluorescent histochemical staining, and we got strong signals in muscle, but scarcely any signal in brain (Fig. 1e). By a histologic HE staining, we observed serious pathological changes in muscle tissue after EV71 infection (Fig. 1f, upper panels). These findings suggest that virus replication mostly occurs in muscle, and relatively low in brain and other organs from this mouse model of EV71 infection. The results from qPCR assay show that the expression of miR-16-5p was upregulated approximately twofold in muscle from EV71-infected mice compared with mock-infected group (Fig. 1d). The upregulation of miR-16-5p by EV71 infection in muscle was further confirmed by *in situ* hybridization (Fig. 1f, lower panels). However, we did not observe significant upregulation of miR-16-5p in brain tissue or any other tissues, except for muscle (Fig. 1d). This may because of there is low virus replication in these tissues as shown in Fig. 1b.

Considering the relatively low virus replication in brain from the EV71-infected mice through intraperitoneal injection, we tried to infect mouse with EV71 directly through intracerebral injection. As shown in Fig. 1g, in brain, the expression of EV71 is about fiftyfold higher in EV71-infected mice through intracerebral injection than the mice through intraperitoneal injection. We further tested the expression of miR-16-5p and found that the expression of miR-16-5p was upregulated in brain from EV71-infected mice through intracerebral injection compared with mock-infected group (Fig. 1h). We also tested the expression of EV71 in cervical spinal cord, and we found that the expression of EV71 in cervical spinal cord was about fivefold higher than that in the extract from whole brain tissue homogenates (Fig. 1i). Through immunofluorescent histochemical staining assay, we observed the expression of EV71 VP1 proteins in cervical spinal cord from the mice infected with EV71through intracerebral injection (Fig. 1k, upper panels). By a histologic HE staining, we also observed pathological changes in cervical spinal cord after EV71 infection (Fig. 1k, lower panels). Meanwhile, the upregulated expression of miR-16-5p by EV71 infection was much obvious in cervical spinal cord than that in whole brain (Fig. 1j). As shown in Fig. 1l and m, we further confirmed EV71-induced upregulation of miR-16-5p in cervical spinal cord by *in situ* hybridization combing with statistical analysis. In summary, these results indicate that the expression of miR-16-5p is upregulated by EV71 infection both *in vitro* and *in vivo*.

### EV71 promotes pri-miR-16-1 processing

**Figure 2:**
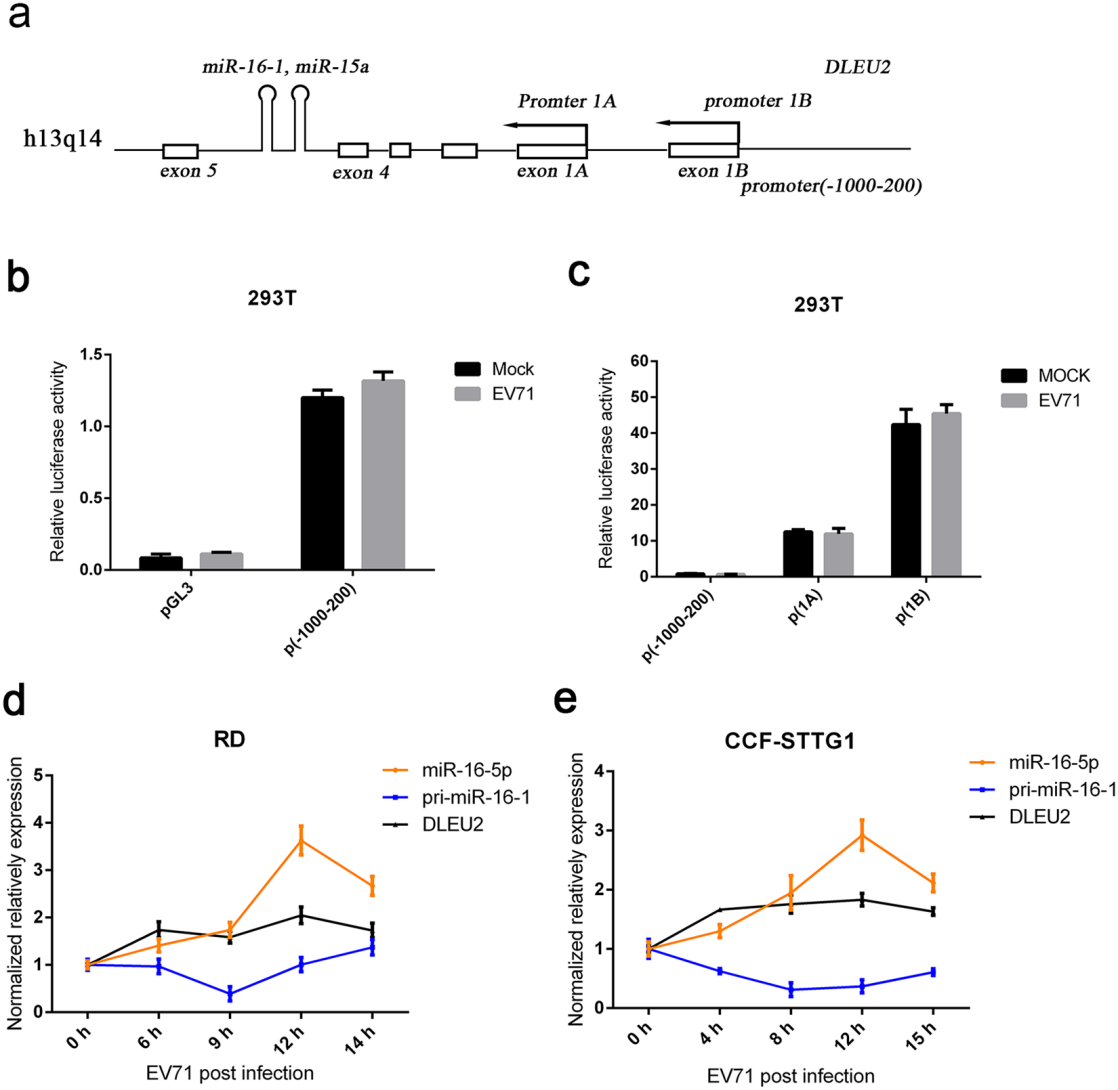
EV71 promotes pri-miR-16-1 processing. (**a**) Schematic of the miR-16-1 gene and its promoter regions. Promoter 1 A and promoter 1B are two previously reported alternative promoters. The promoter (−1000–200) was from 1000 bp upstream to 200 bp downstream of oriented transcriptional start sites. (**b**,**c**) The EV71infection does not influence the transcription of pri-miR-16-1. Promoters 1A and 1B and −1000–100 regions were cloned to pGL3 vectors. Transcription experiments were performed with indicated reports or empty vectors together with an internal control, pRL-TK. Transfected 293 T cells were subjected to EV71 or mock infection for another 12 h. Then, reporter activity was determined by dual luciferase reporter assays. (**d,e**) Expression analysis of pri-miR-16-1 and DLEU2 in EV71-infected RD (**d**) and CCF-STTG1 cells (**e**). After EV71 virus infection for the indicated time, the expression levels of pri-miR-16-1 and DLEU2 were quantified by using qRT-PCR and the expression level of GAPDH as internal control. The relative expression levels of the target genes were calculated according to the 2^−∆∆*Ct*^ method. Data are representative of a minimum of three independent experiments, with each determination performed in triplicate.

Numerous microRNAs are encoded within the introns of other larger coding or non-coding genes with or without their own promoters^51,52^. In the latter case, the expression of the encoded miRNA would depend on the promoter status of the host gene^52^. *miR-15a/16-1* is encoded within an intronic region of the non-coding *Dleu2* gene in both human and mouse and is transcribed off the *Dleu2* promoter^53,54^. To test whether EV71 regulates the transcription of miR-16-1, we performed experiments using luciferase reporter constructs carrying *Dleu2* promoter to assess influence on miR-16-1 transcription by EV71 infection. We cloned 1000 bp up-stream and 200 bp down-stream of the oriented transcriptional start sites, that is, around the putative first exon of DLEU2, into the luciferase reporter plasmid PGL3 (Fig. 2a). The plasmids and empty vector were transfected into 293 T cells, and luciferase activity was measured. The results show that the luciferase activity of *Dleu2* promoter presented minimal change after EV71 infection for 12 h (Fig. 2b). We also cloned the two alternative promoters of *Dleu2* ^55^ into PGL3 and transfected them into 293 T cells. Similar to promoter (−1000–200), the luciferase activity of neither a promoter1A nor a promoter 1B significantly changed after EV71 infection (Fig. 2c). To further confirm the influence of EV71 on the transcription of miR-16-1, the DLEU2 mRNA level was measured in RD and CCF-STTG1 cells after EV71 infection. A modest increase was observed in the expression of DLEU2 following EV71 infection (Fig. 2d and e). Interestingly, we observed that the expression of pri-miR-16-1 decreased at early stages of EV71 infection and then recovered slightly after the expression of miR-16-5p was largely induced (Fig. 2d and e). These results indicate that EV71 probably does not promote the transcription of miR-16-1 gene but the processing of pri-miR-16-1.

### EV71 upregulates miR-16-5p expression through caspase-dependent pathways

**Figure 3:**
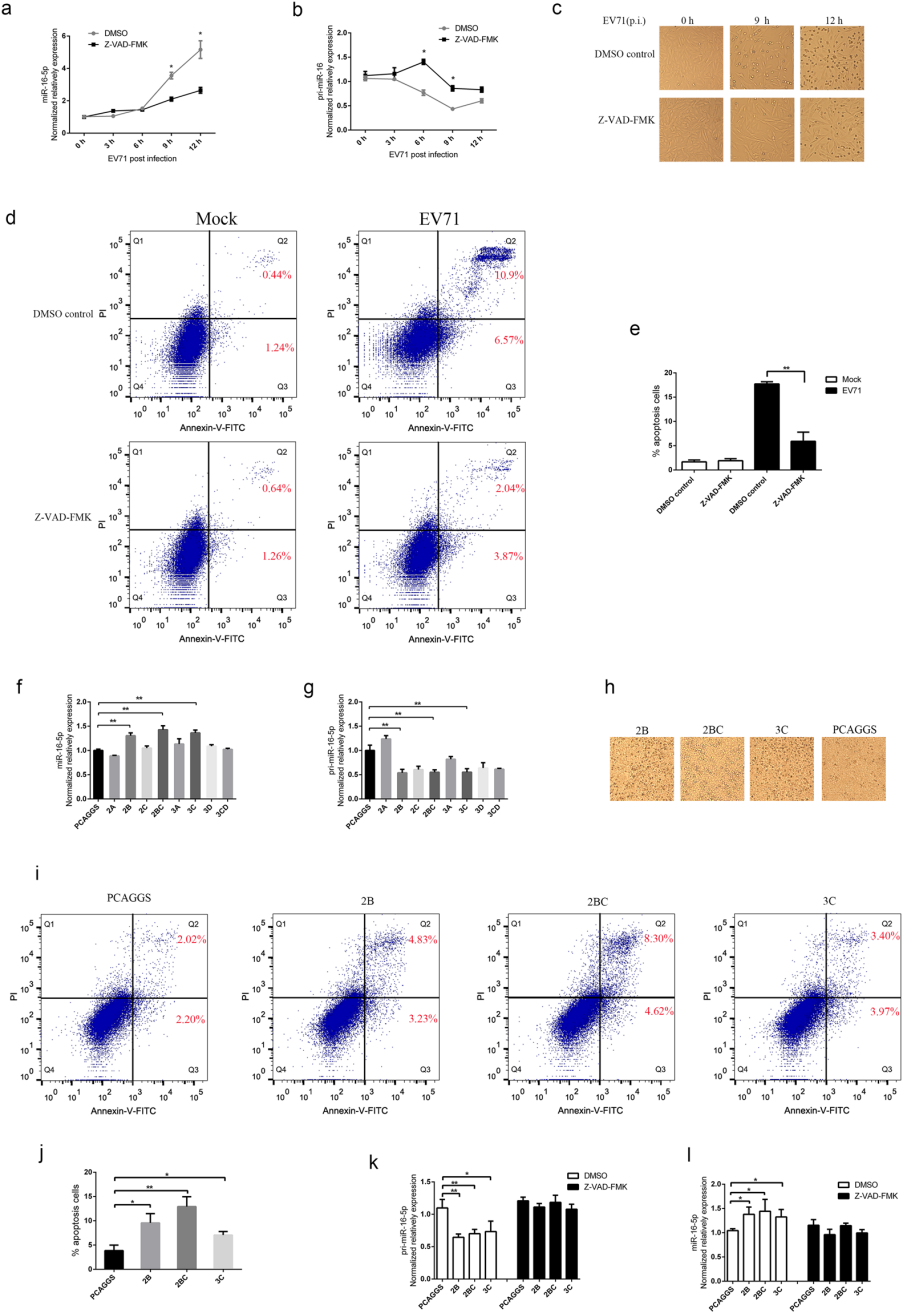
EV71 upregulates the expression of miR-16-5p through caspase-dependent pathways. (**a**,**b**) EV71-induced upregulation of miR-16-5p is inhibited by pan-caspase inhibitor, Z-VAD-FMK. CCF-STGG1 cells were treated with 20 μM Z-VAD-FMK or DMSO as control for 3 h prior to EV71 infection. After EV71 infection for 0, 3, 6, 9 and 12 h, the cells were collected for RNA extraction. The expression levels of miR-16-5p (**a**) and pri-miR-16-1 (**b**) were quantified using qRT-PCR. (**c**) Z-VAD-FMK treatment inhibits EV71-induced CCF-STTG1 apoptosis. CCF-STTG1 cells were pre-treated with Z-VAD-FMK and infected with EV71 as described above. After EV71 infection for 0, 6 and 9 h, cells were observed, and pictures of dishes were obtained using a phase-contrast microscope (Nikon Diaphot 300). (**d**,**e**) Analysis EV71-induced apoptosis in CCF-STTG1 cells using flow cytometry. CCF-STTG1 cells were pre-treated with 20 μM Z-VAD-FMK or DMSO as control for 3 h prior to EV71 infection and infected with EV71. After EV71 infection for 12 h, the cells were harvested and resuspended in binding buffer. The cell suspension was incubated with 5 μL Annexin V-FITC and 10 μL PI for 15 min in a dark place. Then flow cytometry (BD Biosciences, USA) was used to determine apoptosis of the CCF-STTG1 cells (**d**). Annexin V-FITC positive cells were expressed as mean ± SD from three different replicates (**e**). (**f**,**g**) Analysis the influence of EV71 non-structural proteins on the expression of miR-16-5p. 293T cells were transfected with eight non-structure proteins and empty vector PCAGGS. At 24 h post-transfection, cells were collected, and the expression levels of miR-16-5p (**f**) and pri-miR-16-1 (**g**) were examined by qRT-PCR. (**h**,**i** and **j**) EV71 2B, 2BC and 3C proteins can induce cell apoptosis. At 24 h post-transfection with 2B, 2BC, 3C and empty vector PCAGGS, the 293T cells were observed, and pictures of dishes were obtained by using a phase-contrast microscope (**h**). Then the transfected cells were harvested and resuspended for flow cytometry analysis with Annexin V/PI apoptosis assay kits (**i**). Annexin V-FITC positive cells were collected from three different replicates for statistical analysis (**j**). (**k**,**l**) 2B, 2BC, 3C-induced upregulation of miR-16-5p are inhibited by pan-caspase inhibitor, Z-VAD-FMK. 293T cells were transfected with 2B, 2BC, 3C and empty vector PCAGGS respectively. At 24 h post-transfection, cells were collected, and the expression levels of miR-16-5p (**l**) and pri-miR-16-1 (**k**) were examined by qRT-PCR. Data in (**a**,**b**,**e**,**f**,**g**,**j**,**k** and **l**) shown are the means and standard deviations (mean ± SD) and analyzed by Student’s *t* test. Asterisks denote significant differences between indicated samples (*p < 0.05, **p < 0.01).

To test whether EV71-induced apoptosis contributes to the expression of miR-16-5p, we treated CCF-STTG1with Z-VAD-FMK, a cell-permeant pan caspase inhibitor, and detected the expression levels of pri-miR-16-1 and miR-16-5p after EV71 infection. The results showed that the expression of miR-16-5p was evidently inhibited in cells pre-treated with Z-VAD-FMK compared with dimethyl sulphoxide (DMSO) control after 6 hours with EV71 infection (Fig. 3a). Accordingly, the processing of pri-miR-16-1 was slower in cells treated with Z-VAD-FMK than that treated with DMSO control (Fig. 3b). As Fig. 3c shows, pre-treatment of cells with Z-VAD-FMK significantly attenuates EV71-induced cytopathic effect in CCF-STTG1 cells. Furtherly, quantification of apoptotic cells was performed with flow cytometry. The results indicate that the apoptotic cells significantly increased after EV71 infection (Fig. 3d). Both EV71-induced early and late apoptotic cells were obviously inhibited by pre-treatment of cells with Z-VAD-FMK (Fig. 3d and e). These results suggest that caspase-dependent apoptosis induced by EV71 can promote pri-miR-16-1 processing.

To test whether EV71 non-structural proteins could promote the expression of miR-16-5p, we transfected EV71 2A, 2B, 2BC, 3A, 3B, 3C, 3CD and empty vector PCAGGS into 293T cells and used qPCR to detect the expression of pri-miR-16-1 and miR-16-5p. As Fig. 3g shows, the expression of pri-miR-16-1 was lower in cells transfected with the other six non-structural proteins, except for 2A, compared with cells transfected with PCAGGS. However, only 2B, 2BC and 3C were able to promote the expression of miR-16-5p (Fig. 3f). In addition, we tested 2B, 2BC and 3C induced apoptosis through Annexin V/PI assay. The results show that all the three proteins can induce cell apoptosis (Fig. 3h–j).These results indicate that the upregulation of miR-16-5p by EV71 2B, 2BC and 3C may relate to its’ induced apoptosis. Thus, we further tested if the caspase inhibitor, Z-VAD-FMK, could inhibit the upregulation of miR-16-5p by 2B, 2BC and 3C. The results showed that pre-treated the cells with Z-VAD-FMK inhibited the upregulation of miR-16-5p and downregulation of pri-miR-16-1 by all the three proteins (Fig. 3k and l). In summary, these data indicate that EV71-induced apoptosis enhances pri-miR-16-1 processing. Moreover, 2B, 2BC and 3C proteins play an important role in EV71-induced upregulation of miR-16-5p.

### Overexpression of miR-16-5p enhances EV71-induced apoptosis and inhibits virus replication

**Figure 4:**
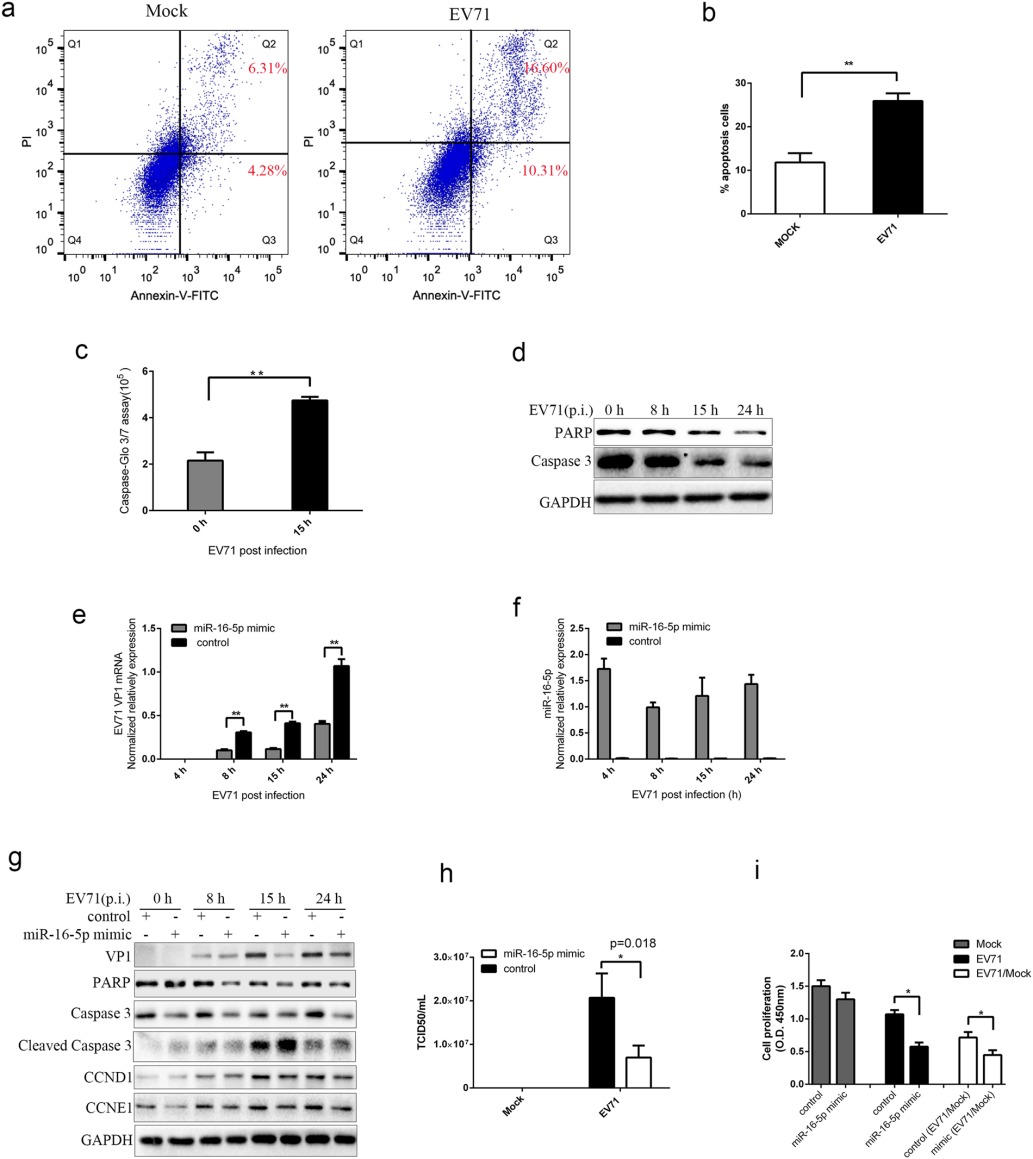
MiR-16-5p enhances EV71-induced apoptosis and inhibits virus replication. (**a**,**b**) Analysis EV71-induced apoptosis in SK-N-SH cells using flow cytometry. 15 h after SK-N-SH cells were infected with EV71, the cells were harvested and analysed by flow cytometry with Annexin V/PI apoptosis assay kits (**a**). Annexin V-FITC positive cells were collected form three different replicates for statistical analysis (**b**). (**c**) Measurement of caspase-3/7 activity in SK-N-SH cells after EV71 infection. SK-N-SH cells were cultured in 96-well plates and infected with EV71 for 0 and 15 h. Caspase-Glo® 3/7 reagent (Promega) were added according the standard protocol, and the luminescence of each sample was measured using a plate-reading luminometer as directed by the luminometer manufacturer. (**d**) Western blot analysis for PARP and Caspase 3 in EV71-infected SK-N-SH cells. Cells were harvested at the indicated times points after EV71 infection. Anti-PARP, anti-Caspase 3 and anti-GAPDH antibodies were used to detect the expression levels of PARP, Caspase 3 and GAPDH, respectively. (**e**,**f**) Overexpression of synthetic miR-16-5p mimics inhibits EV71 replication. SK-N-SH cells transfected with miR-16-5p mimics or control oligonucleotides. Cells were harvested at the indicated times points after EV71 infection. The expression levels of the EV71 VP1 gene were measured by qRT-PCR (**e**). The expression of miR-16-5p was determined both in cells transfected with miR-16-5p mimics and control mimics by using qRT-PCR (**f**). (**g**) Western bolt analysis the influence on EV71 replication and virus-induced apoptosis by overexpressing miR-16-5p. Cells transfected with miR-16-5p mimics or control oligonucleotides were infected with EV71 and assayed for protein expression of EV71 VP1, PARP, caspase 3, cleaved caspase 3, CCND1 and CCNE1. GAPDH was used as a loading control. (**h**) Titration of EV71 in infected cells transfected with miR-16-5p mimics or control oligonucleotides. The transfected SK-N-SH cells were harvested after EV71 infection for 15 h. Virus titters in infected and mock infected cells were determined by TCID50 assays. (**i**) Overexpression of miR-16-5p inhibits cell proliferation in EV71-infected cells. SK-N-SH cells were transfected with miR-16-5p mimics or control oligonucleotides. 36 h after transfection, the cells were infected with EV71 for 15 h. CCK-8 assay was performed to measure proliferation in EV71 infected cells. Data represent the mean ± standard deviation of the optical density (OD) value detected at 450 nm from three independent experiments. Data in panels (**b**,**c**, **e**,**f**,**h** and **i**) are representative of at least three independent experiments, with each determination performed in triplicate (mean ± SD).Asterisks denote significant differences between indicated samples (*p < 0.05, **p < 0.01, Student’s *t* test). The blots in d and g were cropped and the full-length blots were displayed in Supplementary Information file.

EV71 infection can impact the mitochondrial apoptotic pathway and induce apoptosis in numerous cell lines^56,57^. To confirm that EV71 infection can induce apoptosis in SK-N-SH cells, we performed flow cytometry analysis in EV71-infected and mock-infected cells with Annexin V/PI apoptosis detection kits. As Fig. 4a and b shows, the early and late apoptotic cells significantly increased after EV71 infection (Fig. 4a and b). We further assayed the activity of caspase-3 and caspase-7 and the expression of caspase 3 and poly ADP ribose polymerase (PARP) proteins after EV71 infection in SK-N-SH cells. The results from caspase-3/7 activity fluorometric assay show that the enzyme activity of caspase-3/7 is elevated after EV71 infection (Fig. 4c). Western blot tests show that the amount of Caspase-3 and PARP are decreased after EV71 infection (Fig. 4d). These results indicate that EV71 infection can induce SK-N-SH cells apoptosis via the caspase-dependent pathways.

To investigate whether miR-16-5p could influence EV71 replication, we used gain-of-function approaches in SK-N-SH cells, which were transfected with synthetic miR-16-5p mimics, and observed the effects on EV71 replication thereafter. The qPCR assays show that the replication of EV71 is inhibited in SK-N-SH transfected with mimics compared with those transfected with control oligonucleotides (Fig. 4e and f). The western blot assays indicate that the expression of EV71 VP1 proteins in SK-N-SH cells transfected with mimics was lower than those transfected with control oligonucleotides after EV71 infection for 8 h (Fig. 4g). In addition, we also performed TCID50 assays and the results show that it is about threefold reduction in SK-N-SH cells transfected with mimics compared with those transfected with control oligonucleotides (Fig. 4h). These data indicate that upregulation of miR-16-5p expression can inhibit the EV71 replication in SK-N-SH cells.

MiR-16-5p plays a pivotal role in cell cycle regulation by targeting CDK6, CCND1, CCNE1 and CDC7^53^. The expression of miR-16-5p inhibits cell proliferation and promotes apoptosis of cancer cells^58^. To test whether the upregulated miR-16-5p modulates EV71 induced SK-N-SH apoptosis, cells transfected with synthesised miR-16-5p mimics and control oligonucleotides were infected with EV71. The results from western blot assays indicate that overexpressed miR-16-5p promotes the EV71-induced cleavage of caspase-3 and reduction of PARP compared with cells expressed with control oligonucleotides (Fig. 4g). We also detected the expression of CCND1 and CCNE1 which are targeted by miR-16-5p. As Fig. 4g shows, the expression of CCND1and CCNE1are restrained in miR-16-5p transfected cells compared with control cells infected with EV71. What’s more, we performed cell proliferation assays to test if miR-16-5p could influence the proliferation of EV71-infected cells. The results indicate that overexpression of miR-16-5p can inhibit cell proliferation in EV71-infected cells (Fig. 4i). Taken together, these results suggest that miR-16-5p can inhibit the expression of CCND1and CCNE1 and promotes EV71-induced caspase-dependent apoptosis.

**Figure 5:**
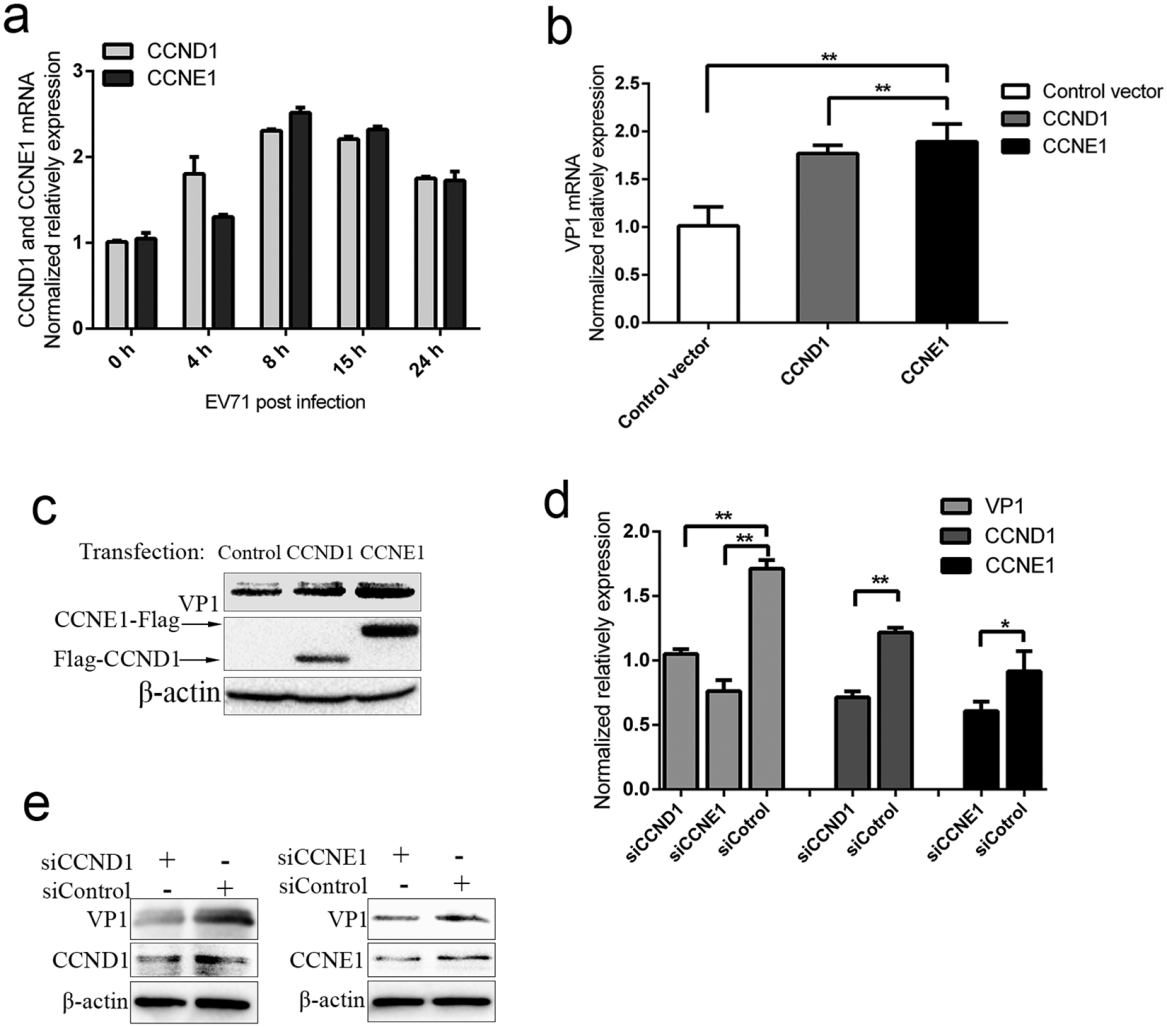
CCND1 and CCNE1 facilitate EV71 replication. (**a**) The expression of CCND1 and CCNE1 were induced by EV71 infection. SK-N-SH cells were harvested after EV71 infection for the indicated times, and the expression levels of CCND1 and CCNE1 were measured by qRT-PCR. (**b**,**c**) Overexpression CCND1 and CCNE1 promote EV71 replication. SK-N-SH cells were transfected with Flag-CCND1, CCNE1-Flag and empty vector PCAGGS. 24 h after transfection, the cells were infected with EV71 for 15 h and then harvested for qRT-PCR (**b**) and western blot (**c**) assays. (**d**,**e**) Knock-down of the expression levels of CCND1 and CCNE1 attenuates EV71 replication. SK-N-SH cells were transfected with control siRNA (NC) or individual*-*specific siRNAs directed against CCND1 or CCNE1. Cells were infected with EV71 for 15 h at 48 h after transfection. Then, the cells were assayed for expression of EV71 genes, CCND1 and CCNE1 by qRT-PCR (**d**) and western blot assay (**e**). Data in panels (**a**,**b** and **d**) are representative of at least three independent experiments, with each determination performed in triplicate (mean ± SD). Asterisks denote significant differences between indicated samples (*p < 0.05, **p < 0.01, Student’s *t* test). The blots in c and e were cropped and the full-length blots were displayed in Supplementary Information file.

Interestingly, we found that EV71 infection enhances the expression levels of CCND1 and CCNE1 (Fig. 5a). Thus, we supposed that if CCND1and CCNE1 could influence EV71 replication. Through qPCR and western blot assays, we found that overexpressed CCND1 and CCNE1 can promote EV71 replication (Fig. 5b and c). Knockdown of CCND1 or CCNE1 with specific siRNAs can inhibit EV71 expression (Fig. 5d and e). These results suggest that CCND1 and CCNE1 contribute to EV71 replication and play an important role in suppression of EV71 replication by miR-16-5p.

### Inhibition of miR-16-5p suppresses EV71-induced apoptosis and facilities virus replication

**Figure 6:**
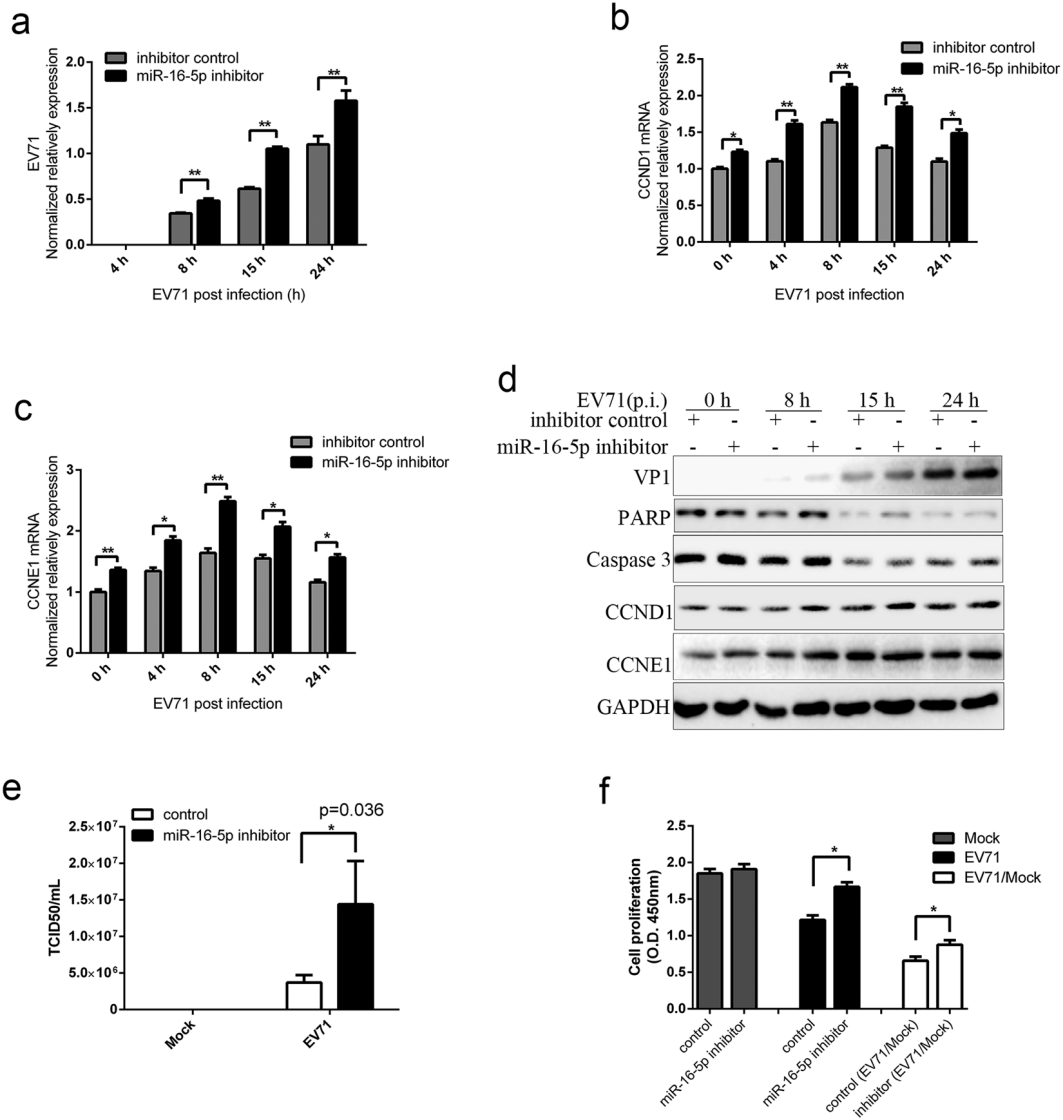
The inhibition of miR-16-5p facilitates EV71 replication and inhibits virus-induced apoptosis. (**a**,**b** and **c**) Inhibition of miR-16-5p promotes the replication of EV71 and expression of CCND1 and CCNE1. SK-N-SH cells transfected with a miR-16-5p inhibitors or control oligonucleotides were infected with EV71. After EV71 infection for 0, 4, 8, 15 and 24 h, cells were assayed for the expression levels of EV71 gene (**a**), CCND1 (**b**) and CCNE1(**c**) by qRT-PCR. (**d**) Western blot analysis the expression of EV71 VP1, PARP, Caspase 3, CCND1 and CCNE1 in SK-N-SH cells transfected with miR-16-5p inhibitors or control oligonucleotides after EV71 infection for the indicated time. (**e**) Titration of EV71 in infected cells transfected with miR-16-5p inhibitors or control oligonucleotides. The transfected SK-N-SH cells were harvested after EV71 infection for 15 h. Virus titters in infected and mock infected cells were determined by TCID50 assays. (**f**) Knock down the expression of miR-16-5p promote cell proliferation in EV71-infected cells. SK-N-SH cells were transfected with miR-16-5p inhibitors or control oligonucleotides. After 48 h after transfection the cells were infected with EV71 for 15 h. CCK-8 assay was performed to measure proliferation in EV71 infected cells. Data represent the mean ± standard deviation of the optical density (OD) value detected at 450 nm from three independent experiments. Data in panels (**a**,**b**,**c**,**e** and **f**) are representative of at least three independent experiments, with each determination performed in triplicate (mean ± SD). Asterisks denote significant differences between indicated samples (*p < 0.05, **p < 0.01; Student’s *t* test for statistical analyses). The blots in d were cropped and the full-length blots were displayed in Supplementary Information file.

To further determine the role of miR-16-5p in EV71 infection, we synthesised miR-16-5p inhibitors, transfected them into SK-N-SH cells and then observed the effects on virus replication and virus-induced apoptosis. As shown in Fig. 6a and d, inhibition of miR-16-5p increases the replication of EV71 genes (Fig. 6a) and the expression of EV71 VP1 proteins (Fig. 6d). The results from TCID50 assays show that there was about threefold increment in miR-16-5p inhibitors transfected cells compared with control inhibitors transfected cells (Fig. 6e). In addition, we found that the amount of Caspase-3 and PARP decreased more slowly in cells transfected with miR-16-5p inhibitors compared with cells transfected with control inhibitors with EV71 infection (Fig. 6d). We also detected the expression of CCNE1 and CCND1. As Fig. 6b,c and d, the expression of CCND1 and CCNE1 at both mRNA (Fig. 6b and c) and protein (Fig. 6d) levels are enhanced in miR-16-5p inhibitors transfected cells compared with inhibitors control transfected cells with EV71infection. The results from cell proliferation assays show that inhibition of miR-16-5p can promote cell proliferation in EV71-infected cells (Fig. 6f). These data indicate that miR-16-5p can inhibit EV71 replication and promote virus-induced caspase-dependent apoptosis.

## Discussion

With deep sequencing technology, we can conveniently obtain miRNA profiles in different samples, such as serum from HMFD and cells or mouse tissues with EV71 infection. This approach is effective to find miRNAs that may be essential in EV71 infection. Several research groups have performed comprehensive miRNA profiling in EV71-infected cells by deep sequencing or microarrays. For example, Cui revealed 64 miRNAs with more than twofold changed expression levels in response to EV71 infection^59^. Using NanoString Counter technology, Robert recently found that 44 miRNAs were observed in patients with EV71 infections with a minimum of twofold elevation and 133 miRNAs with a twofold reduction compared with the same miRNAs in healthy controls^60^. These researches provide a deeper understanding of the mechanisms underlying host-EV71 interaction. However, the specific role of the up- or down-regulated miRNAs in EV71 infection are needed to be further investigated. In our study, we focused on miR-16-5p, a key molecule in cell cycle regulation that is upregulated in HFMD and found that miR-16-5p was largely induced by EV71-induced apoptosis. Furthermore, miR-16-5p can promote EV71-induced nerve cells apoptosis via caspase dependent pathways. We also found that miR-16-5p can inhibit EV71 replication. CCND1 and CCNE1 played an important role in the EV71 replication inhibition by miR-16-5p. These findings would be beneficial to understand the interaction between the virus and the host.

The *miR-15a* and *miR-16-1* cluster is located in the intronic region of *Dleu2* and encodes a long non-coding RNA at the chromosome band 13q14^55,58^. The transcription of *miR-16-1* is controlled by the promoters of *Dleu2* ^61^. In our study, we detected no significant influence on the promoter regions of DLEU2 through luciferase activity assays. These results demonstrate that EV71 may not directly influence the transcription of DLEU2. However, the mRNA level of DELU2 increases about onefold after EV71 infection. Without considering the existence of hitherto undiscovered alternative promoter regions of DLEU2, we speculate that EV71 may influence the stability of DLEU2 mRNA. Interestingly, we found that the expression of miR-16-5p is largely induced by EV71, meaning that EV71 infection promotes the pri-miR-16-1 process into mature miR-16-5p. As the host gene of miR-16-1, the primary transcript of DLEU2 contains the pri-miR-16-1 sequences, which can further be processed to miR-16-5p^55^. The processing of the DLEU2 primary transcript may influence the stability of DLEU2 mRNA.

The targets of miR-16-5p, such as CCNE1 and CCND1, are mostly associated with cell cycle regulations^62^. It has been reportedly that miR-16-6p can inhibit cell proliferation, promote the apoptosis of cancer cells and suppress tumourigenicity both *in vitro* and *in vivo* ^53,54,62,63^. Here, we found that EV71-induced cell apoptosis was promoted by miR-16-5p, whose expression was controlled by the caspase-dependent apoptosis signalling pathway. This promotion forms a positive activation loop in EV71 induced apoptosis reactions. In severe HFMD, we detected higher miR-16-5p expression levels in the exosomes extracted from serum^50^. In addition, we revealed that miR-16-5p promotes EV71-induced apoptosis in nerve cells. Thus, the miR-16-5p-mediated activation loop may play a pivotal role in EV71-induced severe neuropathic diseases. However, in brain, we did not detect an evident increase in miR-16-5p in the mice infected with EV71 through intraperitoneal injection. In our study, we found that the replication of EV71was extremely low in brain tissue. Reportedly, the EV71 virus may exist in particular positions, especially in spinal cord gray matter, brainstem, hypothalamus, and subthalamic and dentate nuclei after virus invades the nerve system^64,65^. Therefore, we supposed that the promotion of miR-16-5p expression by such low EV71 virus may not be significant and may be attenuated when we analysed the extract from the whole murine brain homogenates. To solve the low replication level of EV71 in brain tissues and the difficulty for us to separate different parts of brain from about two weeks old mice, we tried to infect mice with EV71 directly through intracerebral injection. We found that EV71 virus increased about fiftyfold in brain infected with EV71 through intracerebral injection than intraperitoneal injection. In this EV71-infected mouse brain, we observed the up-regulation of miR-16-5p by virus infection. Though the EV71 virus was increased through intracerebral injection, it is still in low level compared with muscle from mice infected EV71through intraperitoneal injection. The cervical spinal cord, in which may exist relatively high EV71 virus and it is easy for us to separate^65^. So, we decided to detect the expression of EV71 and miR-16-5p in cervical spinal cord. The results indicated that the EV71 virus was higher in cervical spinal cord than in whole brain, and the upregulation of miR-16-5p by EV71 was much obviously than in whole brain. These data indicate that EV71 can induce upregulation of miR-16-5p in central nerve system. However, more work is needed to exam the effect of miR-16-5p in central nerve systems with well-studied EV71 infection mouse model.

In contrast to brain, it presents a large number of viruses and a severe cytopathic effect in muscle from our EV71 infection mouse model. In there, we can easily detect that the expression of miR-16-5p is upregulated by EV71. Thus, the miR-16-5p probably contributes to skeletal muscle damage in infected mice and may become the target for drug development.

Apoptosis, or programmed cell death, is not only essential role in the development and the maintenance of homeostasis in multicellular organisms but is also an essential component of cell response to injury^66^. In particular, cells will undergo programmed cell death following viral infection, which s may abort the production and release of progeny virus^67^. Most viruses, including EV71, can trigger the apoptosis or programmed cell death of the infected cell. To facilitate virus replication, some virus can prevent the premature death of host cell during lytic infection, and certain viruses seem to use apoptosis as a mechanism of cell killing and virus spread during latent infections^67^. Here, miR-16-5p acts as a monitor of EV71 infection, promoting the programmed cell death of the infected cell and inhibiting virus replication. In our study, we found that CCND1 and CCNE1, whose expression levels increased after EV71 infection, are able to promote EV71 replication. CCND1 and CCNE1 are the main regulators for the transition from G_1_ to S phase, which determines cell division^68^. Thus, EV71 may delay virus-induced apoptosis by upregulating the expression levels of CCNE1 and CCND1. On the contrary, miR-16-5p targets CCND1 and CCNE1 to promote apoptosis to abort virus replications. Here, miR-16-5p is not only a bio-marker in hand-foot-mouth diseases but is an anti-viral regulator in virus infection.

In conclusion, we showed that miR-16-5p can act as a positively regulator in EV71-induced apoptosis and can be largely induced by EV71 infection. Moreover, miR-16 can inhibit virus replication by targeting the cell cycle regulating genes CCND1 and CCNE1, which promote virus induced apoptosis. The data help explain the upregulated expression of miR-16-5p in EV71 infected children and offer a novel therapy to inhibit EV71 infection.

## Methods

### Synthetic oligonucleotides, antibodies and chemicals

Synthetic oligonucleotides representing the miR-16-5p mimics, the negative control for mimics, the miR-16-5p inhibitor (antisense oligonucleotides to the mature has-miR-16-5p according to sequences in the miRBase) and the negative control for the inhibitor all were obtained from RiBo Biotech (Guangzhou, China). Negative control sequences are based on *C. elegans* miRNA cel-miR-67-3p. The sequence of miR-16-5p mimics was uagcagcacguaaauauuggcg. The sequence of miR-16-5p inhibitors was aucgucgugcauuuauaaccgc. Non-specific control (NC), CCND1 and CCNE1 siRNA were chemically synthesised by GenePharma (Suzhou, China). The sequences are as follows, NC: UUCUCCGAACGUGUCACGUTT; CCND1: GGAGAACAAACAGAUCAUCTT; CCNE1: UGGCCAAAAUCGACAGGACTT. The Digoxigenin (DIG)-labelled miR-16-5p probes to detect miR-16-5p for miRNA *in situ* hybridisation were purchased from Sangon Biotech (Shanghai, China). The probe sequences of probe were CGCCAATATTTACGTGCTGCTA. The EV71 VP1 antibody was obtained from Millipore (Billerica, MA, USA). Mouse anti-GAPDH, mouse anti-CCND1 and rabbit CCNE1 antibodies were acquired from Proteintech (Wuhan, China). Mouse anti-PARP, rabbit anti-caspase 3 and rabbit anti-cleaved caspase 3 were purchased from Cell Signalling Technology (Danvers, MA, USA). The pan-caspase inhibitor Z-VAD-FMK was from Promega Corporation (Wisconsin, USA).

### Plasmid Constructions

To generate luciferase reporter plasmids of the pri-miR-16-1 promoter regions, we amplified the promoter regions of DLEU2 from SK-N-SH genome by using the primers listed below. Primers used for PCR for amplification and cloning the fragments from 1000 bp upstream to 200 bp downstream of the transcription start site 1 (TSS) were (p-1000-200) forward primer F (5′-CGGggtaccAAAGGTGCGACGCGCTTCTTGCCCT -3′) and (p-1000-200) reverse primer R (5′-CCCaagcttTGCGAAAAGGAGAAGGCGGAGCGGT-3′). The primers for amplifying DLEU2 1 A promoter fragments were F (5′-GGggtaccAAGCCGGCAGGGCGGTTTT-3′) and R (5′-CCCaagcttTACCGACTGCGCCAGCCTTG-3′). The primers for amplifying the DLEU2 1B promoter fragments were F (5′-GGggtaccAGATCCTCGTCGGGTGGCG-3′) and R (5′-CCCaagcttTGTGCAGTTTCAGCAAAGCTCCGAGG-3′). The PCR fragments were cloned into pGL3 luciferase reporter vectors (Promega), which were digested with KpnI and HindIII. The sequences of the cloned fragments were verified in its entire length by sequencing.

### Cell culture and transfection

The human embryonic kidney 293T cells were cultured in Dulbecco’s modified Eagle’s medium (DMEM) (Life Technologies) supplemented with 10% (vol/vol) foetal bovine serum (FBS) (Gibco). Neuroblastoma SK-N-SH cells and rhabdomyosarcoma RD cells were obtained from ATCC and cultured in minimum essential mediaHE (Life Technologies) supplemented with 10% (vol/vol) FBS (Gibco). The Human astrocytoma CCF-STTG1 cells were cultured in 1640 (Life Technologies) supplemented with 10% (vol/vol) heat-inactivated FBS (Gibco). All of above cells were cultured at 37 °C and under 5% CO_2_ and 95% air and passaged upon reaching 80–90% confluence. One day before transfection, 293T cells were plated in the appropriate culture dish. When the plated cells reached approximately 60–70% confluence, the cells were transfected with a total of 2 µg plasmid per one well of six-well plates by using calcium phosphate of ProFection (Promega) according to the manufacturer’s instructions. Lipofectamine™ 3000 (Invitrogen) was used for the transfection of SK-N-SH cells according to the manufacturer’s instructions. In general, the SK-N-SH cells should reach about 70–80% confluence when transfected with synthetic miR-16-5p mimics and approximately 30–50% confluence when transfected with synthetic miR-16 inhibitor oligonucleotides.

### Virus propagation, virus titration and infection

Virus propagation. Human EV71 stocks were generated in Vero cells. In brief, Vero cells were seeded in 10 cm^2^ dishes one day before infection. EV71 virus were diluted in DMEM and inoculated onto approximately 70% confluence Vero cells. After adsorption for 1.5 h at 37 °C in a 5% CO_2_ humidified incubator, DMEM supplemented with 2% FBS was added and the infection was allowed to proceed until the monolayer appeared to be completely involved with cytopathic effect (CPE), at one to two days post-infection. The cells and culture medium were collected in 50 mL conical polypropylene tubes and subjected to three freeze–thaw cycles. The suspension was clarified by centrifugation at 4500 rpm for 10 min. The supernatant was transferred into cryovials and stored at −80 °C.

Virus titration. For all viruses stocks, the 50% tissue culture infectious dose (TCID_50_/mL) titres were determined. In brief, 5000 RD cells were seeded in 96-well plates the day before infection. The virus samples were serially diluted with DMEM containing 2% FBS (10^3^ to 10^10^) and then each of dilution were added in wells separately. The plates were incubated at 37 °C in 5% CO2 for 2-5 days. CPE was observed under the microscope after 2 to 5 days. Determination of virus titter, expressed as the 50% tissue culture infectious dose (TCID50), was performed using the Reed-Münch endpoint calculation method.

Virus infection. RD, CCF-STTG1 and SK-N-SH were infected with EV71 virus according to the procedures below. In brief, subconfluent monolayers of RD cells, CCF-STTG1 and SK-N-SH cells grown in 35 mm culture dishes were inoculated with EV71 (BrCr strain) viruses at a multiplicity of infection ranging from 1 to 10 TCID_50_/cell. After a 1.5 h adsorption at 37 °C in a 5% CO_2_ humidified incubator, the virus was removed, and cells were washed with corresponding culture medium without FBS to remove unbound virus before the addition of fresh culture medium supplemented with 10% FBS. The cells were collected for RNA extraction or Western blot analysis after incubation for the indicated times.

### Animal Models

All studies were conducted in strict accordance with the institutional guidelines for animal research and approved by the Administration of Affairs Concerning Experimental Animals of the People’s Republic of China. All animal treatments were reviewed and approved in advance by the Ethics Committee of the Animal House facility of the Wuhan Institute of Virology, Chinese Academy of Sciences, (Wuhan, china) ((protocol number: WIVA07201601; approval date September 1, 2016).

KM, ICR pregnant mice were purchased and used in accordance with the Regulations for the Administration of Affairs Concerning Experimental Animals of the People’s Republic of China. All mice were housed under specific-pathogen-free conditions in individually ventilated caging systems. Two-week-old mice were infected with EV-A71 GZ-CII at the dose of 10^5^ TCID_50_/mouse via intraperitoneal route or at the dose of 10^3^ TCID_50_/mouse via intracerebral route. Body weight and disease manifestations were monitored each day post-infection. The mice were sacrificed after three or four days when oblivious posterior paralysis was observed, and the whole blood, brain, heat, liver, spleen, lung, kidney, small intestine and muscle tissue of mice were collected for RNA extraction. EV71-infected or mock-infected brain and muscle tissue of mice were harvested for immunohistochemistry and *in situ* hybridisation.

### Luciferase assays

Luciferase assays were performed using the dual-luciferase reporter assay system kit (Promega, Madison, WI, USA) according to the manufacturer’s protocol. In brief, 293T cells grown to 60%–70% confluence in 12-well plates and co-transfected with 500 ng of luciferase reporter plasmid containing the promoter regions of DLEU2 (luciferase reporter) and 100 ng of pRL-TK (Renilla luciferase, internal control) with using ProFection calcium phosphate reagents according to the manufacturer’s instruction (Promega). At 24 h post-transfection, the cells were infected with EV71 virus or Sendai virus (SeV) for the indicated durations. Mock-infected cells served as controls. Then, the cells were collected and washed once with cold phosphate-buffered saline (PBS). Passive lysis buffer (Promega) was then added to the cells. After 10–15 min, supernatants were collected following centrifugation at 12,000 × *g* for 30 s, and relative luciferase activity was measured with Dual-Luciferase Reporter Assay System according to the manufacturer’s instruction (Promega).

### Immunohistochemistry

For immunohistochemical assay, whole brain, cervical spinal cord and muscle tissues were blocked in paraffin and subsequently cut into 5 μm sections. After deparaffinisation and rehydration, sections were incubated in Tris-buffered saline (TBS) containing 0.1 Trition for 20 min at room temperature (RT) and then washed for 10 min in TBS. To quench the endogenous peroxidase, we treated sections with 2% H_2_O_2_ in methanol for 20 min. Then, the sections were blocked in TBS containing 10% normal rabbit serum (GIBCO Invitrogen) and 0.2% BSA for 30 min with shaking at RT. Following blocking, brain and muscle sections were incubated with a mouse anit-EV71 VP1 antibody (dilution 1:200) overnight at 4 °C. On the next day, sections were washed in TBS and incubated with the appropriate Texas Red -labelled secondary antibodies (Invitrogen) 1:500 for 1 h and at RT. Sections were washed in TBS, counterstained with DPAI (Sigma-Aldrich) for 8 min at RT, washed in TBS and embedded in Mowiol (Sigma-Aldrich). Slides were then cover-slipped with Vectashield Mounting Medium (Vector Laboratories), and visualised under a fluorescent microscope.

### ***In situ*** hybridization

*In situ* hybridisation was performed as follows. In brief, tissue sections were post-fixed with 4% PFA in PBS (pH7.4) for 20 min at RT. Sections following were acetylated with 0.25% acetic anhydride in 0.1 M trietheanolamine and 0.06% HCl for 10 min at RT to chance tissue penetration and decreases non-specific background staining. After pre-hybridisation for 2 h at RT, sections were incubated with 500 µg/mL of denatured DIG-labeled probe diluted in hybridisation buffer (50% formamide, 5× Denhardt’s solution and 5× SSC, 250 mg/mL baker’s yeast tRNA and 500 mg/mL sonicated salmon sperm DNA) for 15 h at 68 °C. Then, the sections were first washed briefly in 2× SSC followed by incubation in 0.2× SCC for 2 h at 68 °C. Sections were adjusted to RT in 0.2× SSC for 5 min. The DIG-labeled RNA hybrids were detected. Anti-DIG fab fragments conjugated to AP (Boehringer) and diluted 1:2500 in Tris-buffered saline (TBS, pH 7.4) overnight at 4 °C. Binding of the AP-labeled antibody was visualised by incubating the sections in detection buffer (100 Mm Tris-HCl, pH 9.5, 100 mM NaCl and 50 mM MgCl_2_) containing 240 mg/mL levamisole and nitroblue tetrazolium chloride/5-bromo-4-chloro-3-indolyl-phosphatase (Roche) for 14 h at RT. Sections subjected to the entire *in-situ* hybridization procedure, but without any added probe, did not exhibit specific hybridisation signals. In the case of nuclear staining, sections were incubated with Nuclear fast red for 5 min at RT. Staining was visualised using a Nikon ECLIPSE CI microscope. Image analyses were undertaken using Aperio ImageScope Software (v10) and the Positive Pixel Count V9 algorithm (default settings).

### Western blot analysis

Western blot was performed as previously described^69,70^. In brief, whole-cell lysates were generated using Cell lysis buffer (Beyotime Institute of Biotechnology, China) according to the manufacturer’s instructions. After electrophoresis, proteins were transferred for 2.5 h at 4 °C to PVDF membranes (Millipore) in a buffer containing 30 mM Tris, 200 mM glycine and 20% (vol/vol) methanol. Membranes were incubated with primary antibodies overnight at 4 °C. After a standard washing cycle with TBS containing 0.1% Tween 20, membranes were incubated with HRP-conjugated secondary antibody (Pierce) for 2 h at room temperature. After washes, membranes were incubated with immobilon western chemiluminescent HRP substrate (Millipore) followed by analysis using Bio-Red Imaging System.

### RNA isolation and real-time PCR analysis

Total RNA was extracted using the Trizol reagent (Invitrogen) according to the manufacturer’s standard protocol. The first cDNA strand was generated using a random primer and Moloney Murine Leukemia Virus Reverse Transcriptase (Promega). Real-time PCR was conducted with SYBR Green Master Mix (Bio-Rad) on the CFX96 touch real*-*time PCR detection system (Bio-Rad). GAPDH mRNA was measured as a control for the expression of level of DLEU2, pri-miR-16-1, CCND1, CCNE1 and EV71 genomic RNA. U6 rRNA serves as internal control for the expression level of hsa-miR-16-5p.The specific primer sequences are as follows: pri-miR-16-1, F (CCTTGGAGTAAAGTAGCAGCACATAATG), R (ATATACATTAAAACACAACTGTAGAGTATG); DLEU2, F(TGAAGATGTCTTTTGAAAGGTGTAC), R (ACTTTTTCCATGAGGAGGTACAGT); CCND1, F (CTGTGCATCTACACCGACAACT), R (GCATTTTGGAGAGGAAGTGTTC); CCNE1, F (TGTGTCCTGGATGTTGACTGCC), R (CTCTATGTCGCACCACTGATACC); GAPDH, F (GTCTCCTCTGACTTCAACAGCG), R (ACCACCCTGTTGCTGTAGCCAA); EV71 (BrCr), F(GAGAGTTCTATA GGGGACAGT), R(AGCTGTGCTATGTGAATTAGGAA); EV71 (GZ-CII), F (GATAGGGTGGCAGATGTAAT), R (CACAGCGTGTCTCAATCA) and mouse GAPDH, F(AACGACCCCTTCATTGAC), R (TCCACGACATACTCAGCAC). Hsa-miR-16-5p specific bulge-loop miRNA qRT-PCR primers were purchased from Ribo Biotechnology (Guangzhou, China). Data analysis was performed using the 2^−ΔΔ*Ct*^ method.

### Cell apoptosis analysis

Cell apoptosis was analysed with FITC Annexin V Apoptosis Detection kit with PI (Biolgend, USA) according to the manufacturer’s standard protocol. Briefly, the virus-infected cells and mock-infected cells were washed twice with cold BioLegend’s Cell Staining Buffer, and then resuspended in Annexin V Binding Buffer at an appropriate concentration (10^6^ cells/mL). 5 μL of FITC Annexin V and 10 μL Propidium Iodide were added in 100 μL of cell suspension, and then the cells incubated for 15 min at room temperature. After incubation, 400 μL of Annexin V Binding Buffer was added to each tube and the cells were analysed by flow cytometry (BD Biosciences, USA). Annexin V-FITC positive cells from three different replicates were collected for apoptosis index analysis.

### Cell proliferation

Cell Counting Kit-8 (CCK-8) assay were performed to detect cell proliferation. Briefly, after for indicated treatments, the cells were cultured in fresh medium mixed with CCK-8 (10:1) (Dojindo, Shanghai, China) for 2 hours. Then the absorbance was measured with a microplate reader at 450 nm.

### Statistical Analysis

All experiments were reproducible and carried out in triplicate. Data are represented as mean ± SD when indicated and Student’s t test was used for all statistical analyses with the GraphPad Prism 6.0 software. Differences were considered significant when p value was less than 0.05.

## Electronic supplementary material

Supplementary Information

## References

[1] Wang Q (2014). Clinical features of severe cases of hand, foot and mouth disease with EV71 virus infection in China. Arch Med Sci.

[2] Chatproedprai S (2010). Clinical and molecular characterization of hand-foot-and-mouth disease in Thailand, 2008-2009. Jpn J Infect Dis.

[3] Solomon T (2010). Virology, epidemiology, pathogenesis, and control of enterovirus 71. Lancet Infect Dis.

[4] Chumakov M (1979). Enterovirus 71 isolated from cases of epidemic poliomyelitis-like disease in Bulgaria. Arch Virol.

[5] Nagy G, Takatsy S, Kukan E, Mihaly I, Domok I (1982). Virological diagnosis of enterovirus type 71 infections: experiences gained during an epidemic of acute CNS diseases in Hungary in 1978. Arch Virol.

[6] Hamaguchi T (2008). Acute encephalitis caused by intrafamilial transmission of enterovirus 71 in adult. Emerg Infect Dis.

[7] McMinn P, Stratov I, Nagarajan L, Davis S (2001). Neurological manifestations of enterovirus 71 infection in children during an outbreak of hand, foot, and mouth disease in Western Australia. Clin Infect Dis.

[8] Ooi MH, Wong SC, Lewthwaite P, Cardosa MJ, Solomon T (2010). Clinical features, diagnosis, and management of enterovirus 71. Lancet Neurol.

[9] Chan LG (2000). Deaths of children during an outbreak of hand, foot, and mouth disease in sarawak, malaysia: clinical and pathological characteristics of the disease. For the Outbreak Study Group. Clin Infect Dis.

[10] Schmidt NJ, Lennette EH, Ho HH (1974). An apparently new enterovirus isolated from patients with disease of the central nervous system. J Infect Dis.

[11] Huang CC (2001). Neurologic complications of enterovirus 71 infection in children: lessons from this Taiwan epidemic. Acta Paediatr Taiwan.

[12] Lee MS (2010). An investigation of epidemic enterovirus 71 infection in Taiwan, 2008: clinical, virologic, and serologic features. Pediatr Infect Dis J.

[13] Mao QY, Wang Y, Bian L, Xu M, Liang Z (2016). EV71 vaccine, a new tool to control outbreaks of hand, foot and mouth disease (HFMD). Expert Rev Vaccines.

[14] Lee KY (2016). Enterovirus 71 infection and neurological complications. Korean J Pediatr.

[15] He L, Hannon GJ (2004). MicroRNAs: small RNAs with a big role in gene regulation. Nat Rev Genet.

[16] Kim VN, Han J, Siomi MC (2009). Biogenesis of small RNAs in animals. Nat Rev Mol Cell Biol.

[17] Trobaugh DW, Klimstra WB (2017). MicroRNA Regulation of RNA Virus Replication and Pathogenesis. Trends Mol Med.

[18] Londin E (2015). Analysis of 13 cell types reveals evidence for the expression of numerous novel primate- and tissue-specific microRNAs. Proc Natl Acad Sci USA.

[19] Ludwig N (2016). Distribution of miRNA expression across human tissues. Nucleic Acids Res.

[20] Wahid F, Shehzad A, Khan T, Kim YY (2010). MicroRNAs: synthesis, mechanism, function, and recent clinical trials. Biochim Biophys Acta.

[21] Rajewsky N (2006). microRNA target predictions in animals. Nat Genet.

[22] Macfarlane LA, Murphy PR (2010). MicroRNA: Biogenesis, Function and Role in Cancer. Curr Genomics.

[23] Bartel DP (2004). MicroRNAs: genomics, biogenesis, mechanism, and function. Cell.

[24] Kim VN (2005). Small RNAs: classification, biogenesis, and function. Mol Cells.

[25] Lee Y (2004). MicroRNA genes are transcribed by RNA polymerase II. EMBO J.

[26] Lee Y (2003). The nuclear RNase III Drosha initiates microRNA processing. Nature.

[27] Han J (2004). The Drosha-DGCR8 complex in primary microRNA processing. Genes Dev.

[28] Denli AM, Tops BB, Plasterk RH, Ketting RF, Hannon GJ (2004). Processing of primary microRNAs by the Microprocessor complex. Nature.

[29] Nakielny S, Dreyfuss G (1999). Transport of proteins and RNAs in and out of the nucleus. Cell.

[30] Yi R, Qin Y, Macara IG, Cullen BR (2003). Exportin-5 mediates the nuclear export of pre-microRNAs and short hairpin RNAs. Genes Dev.

[31] Ketting RF (2001). Dicer functions in RNA interference and in synthesis of small RNA involved in developmental timing in C. elegans. Genes Dev.

[32] Knight SW, Bass BL (2001). A role for the RNase III enzyme DCR-1 in RNA interference and germ line development in Caenorhabditis elegans. Science.

[33] Chendrimada TP (2005). TRBP recruits the Dicer complex to Ago2 for microRNA processing and gene silencing. Nature.

[34] Gregory RI, Chendrimada TP, Cooch N, Shiekhattar R (2005). Human RISC couples microRNA biogenesis and posttranscriptional gene silencing. Cell.

[35] Guo YE, Steitz JA (2014). Virus meets host microRNA: the destroyer, the booster, the hijacker. Mol Cell Biol.

[36] Mouillet JF, Ouyang Y, Bayer A, Coyne CB, Sadovsky Y (2014). The role of trophoblastic microRNAs in placental viral infection. Int J Dev Biol.

[37] Roberts AP, Lewis AP, Jopling CL (2011). The role of microRNAs in viral infection. Prog Mol Biol Transl Sci.

[38] Tahamtan A (2016). The role of microRNAs in respiratory viral infection: friend or foe?. Rev Med Virol.

[39] Ding SW, Voinnet O (2007). Antiviral immunity directed by small RNAs. Cell.

[40] Maillard PV (2013). Antiviral RNA interference in mammalian cells. Science.

[41] Shimakami T (2012). Stabilization of hepatitis C virus RNA by an Ago2-miR-122 complex. Proc Natl Acad Sci USA.

[42] Luna JM (2015). Hepatitis C virus RNA functionally sequesters miR-122. Cell.

[43] Zheng Z (2013). Human microRNA hsa-miR-296-5p suppresses enterovirus 71 replication by targeting the viral genome. J Virol.

[44] Wen BP, Dai HJ, Yang YH, Zhuang Y, Sheng R (2013). MicroRNA-23b inhibits enterovirus 71 replication through downregulation of EV71 VPl protein. Intervirology.

[45] Ho BC (2014). Inhibition of miR-146a prevents enterovirus-induced death by restoring the production of type I interferon. Nat Commun.

[46] Wu S (2013). miR-146a facilitates replication of dengue virus by dampening interferon induction by targeting TRAF6. J Infect.

[47] Sharma N, Verma R, Kumawat KL, Basu A, Singh SK (2015). miR-146a suppresses cellular immune response during Japanese encephalitis virus JaOArS982 strain infection in human microglial cells. J Neuroinflammation.

[48] Friedlander MR (2008). Discovering microRNAs from deep sequencing data using miRDeep. Nat Biotechnol.

[49] Yin L (2015). Discovering novel microRNAs and age-related nonlinear changes in rat brains using deep sequencing. Neurobiol Aging.

[50] Jia HL (2014). MicroRNA expression profile in exosome discriminates extremely severe infections from mild infections for hand, foot and mouth disease. BMC Infect Dis.

[51] Morlando M (2008). Primary microRNA transcripts are processed co-transcriptionally. Nat Struct Mol Biol.

[52] Rodriguez A, Griffiths-Jones S, Ashurst JL, Bradley A (2004). Identification of mammalian microRNA host genes and transcription units. Genome Res.

[53] Aqeilan RI, Calin GA, Croce CM (2010). miR-15a and miR-16-1 in cancer: discovery, function and future perspectives. Cell Death Differ.

[54] Ofir M, Hacohen D, Ginsberg D (2011). MiR-15 and miR-16 are direct transcriptional targets of E2F1 that limit E2F-induced proliferation by targeting cyclin E. Mol Cancer Res.

[55] Lerner M (2009). DLEU2, frequently deleted in malignancy, functions as a critical host gene of the cell cycle inhibitory microRNAs miR-15a and miR-16-1. Exp Cell Res.

[56] Chang SC, Lin JY, Lo LY, Li ML, Shih SR (2004). Diverse apoptotic pathways in enterovirus 71-infected cells. J Neurovirol.

[57] Chen TC, Lai YK, Yu CK, Juang JL (2007). Enterovirus 71 triggering of neuronal apoptosis through activation of Abl-Cdk5 signalling. Cell Microbiol.

[58] Klein U (2010). The DLEU2/miR-15a/16-1 cluster controls B cell proliferation and its deletion leads to chronic lymphocytic leukemia. Cancer Cell.

[59] Cui L (2010). Identification of microRNAs involved in the host response to enterovirus 71 infection by a deep sequencing approach. J Biomed Biotechnol.

[60] Wang RY, Weng KF, Huang YC, Chen CJ (2016). Elevated expression of circulating miR876-5p is a specific response to severe EV71 infections. Sci Rep.

[61] Calin GA (2008). MiR-15a and miR-16-1 cluster functions in human leukemia. Proc Natl Acad Sci USA.

[62] Liu Q (2008). miR-16 family induces cell cycle arrest by regulating multiple cell cycle genes. Nucleic Acids Res.

[63] Lezina L (2013). miR-16 and miR-26a target checkpoint kinases Wee1 and Chk1 in response to p53 activation by genotoxic stress. Cell Death Dis.

[64] Lee TC (2009). Diseases caused by enterovirus 71 infection. Pediatr Infect Dis J.

[65] McMinn P (2001). Phylogenetic analysis of enterovirus 71 strains isolated during linked epidemics in Malaysia, Singapore, and Western Australia. J Virol.

[66] Elmore S (2007). Apoptosis: a review of programmed cell death. Toxicol Pathol.

[67] Barber GN (2001). Host defense, viruses and apoptosis. Cell Death Differ.

[68] Blomen VA, Boonstra J (2007). Cell fate determination during G1 phase progression. Cell Mol Life Sci.

[69] Liu Q (2016). Human Bocavirus NS1 and NS1-70 Proteins Inhibit TNF-alpha-Mediated Activation of NF-kappaB by targeting p65. Sci Rep.

[70] Zheng C (2015). IFIT5 positively regulates NF-kappaB signaling through synergizing the recruitment of IkappaB kinase (IKK) to TGF-beta-activated kinase 1 (TAK1). Cell Signal.

