# Supplementary material for: MiR-16-5p mediates a positive feedback loop in EV71-induced apoptosis and suppresses virus replication
Source: Sci Rep. 2017 Nov 27;7:16422. doi: 10.1038/s41598-017-16616-7 (PMC5703983; doi:10.1038/s41598-017-16616-7)

MiR-16-5p mediates a positive feedback loop in EV71-induced apoptosis and suppresses virus replication

Caishang Zheng[1, 2], Zhenhua Zheng[2, *], Jianhong Sun[2], Yuan Zhang[2], Chunyu Wei[2], Xianliang Ke[1, 2], Yan Liu[2], Li Deng[1, *], Hanzhong Wang[2]

SUPPLEMENTARY INFORMATION

This file contains the full unedited gel for figure 4d, figure 4g, figure 5c, figure 5e and figure 6d.

Full unedited gel for figure 4d

The red marks indicate the bands reported in Figure 4d.

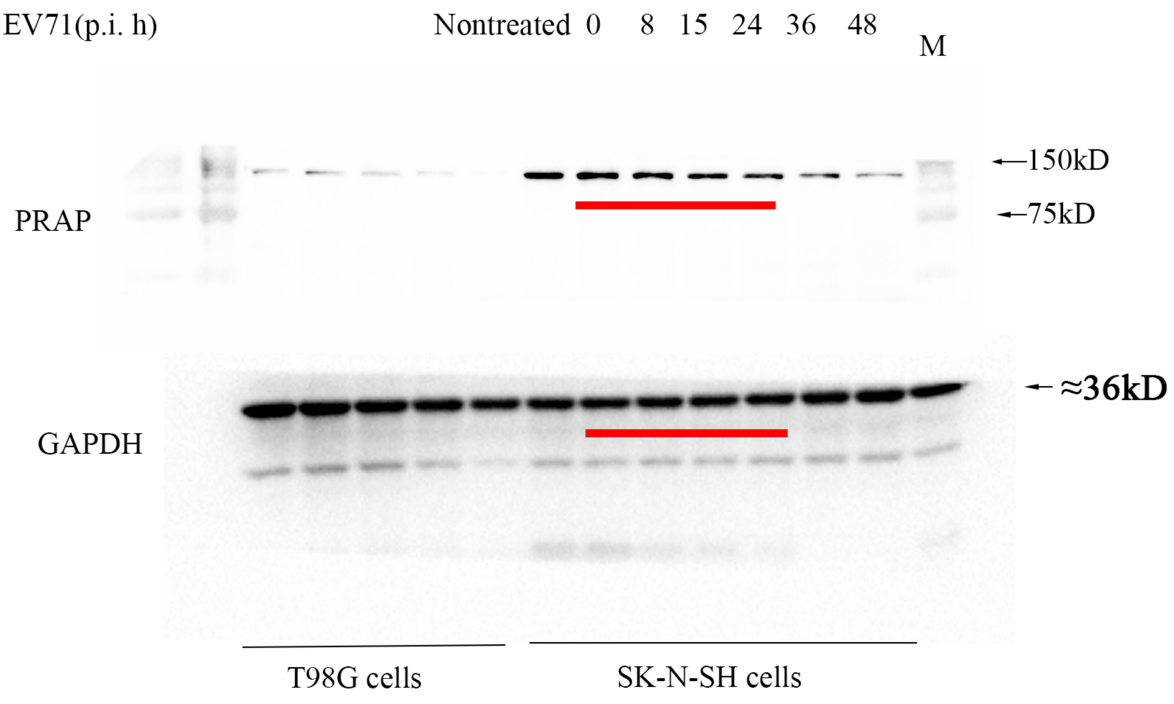

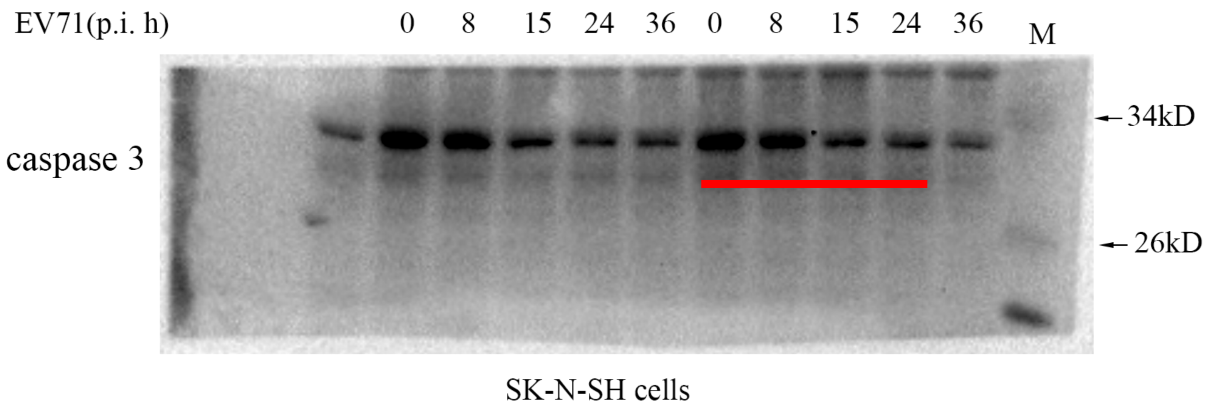

Full unedited gel for figure 4g

The red marks indicate the bands reported in Figure 4g.

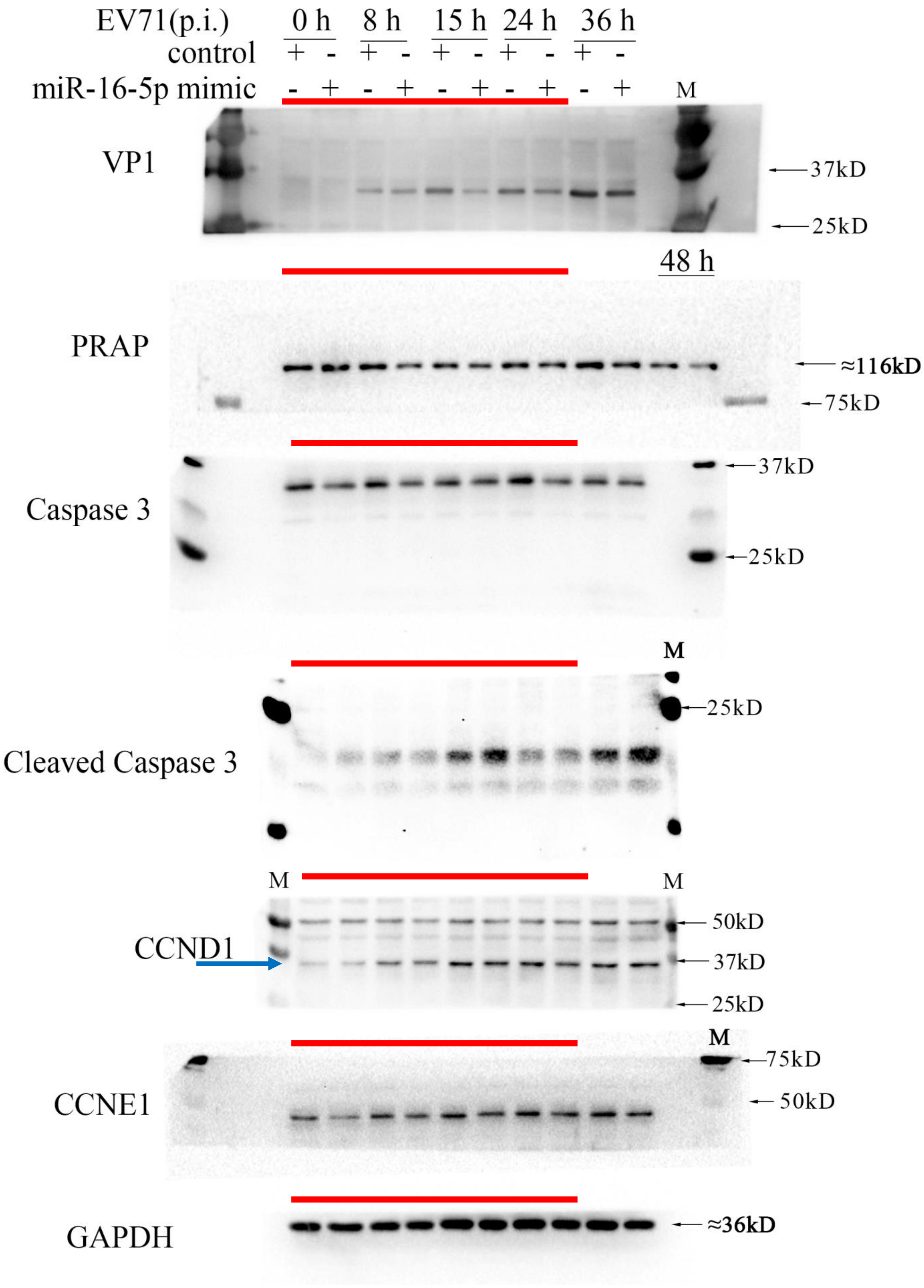

Full unedited gel for figure 5c

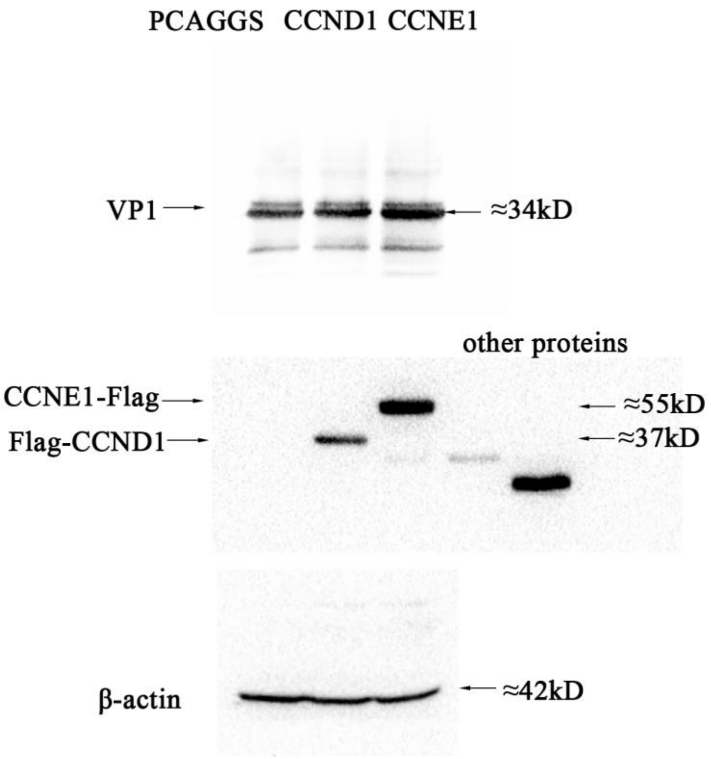

Full unedited gel for figure 5e

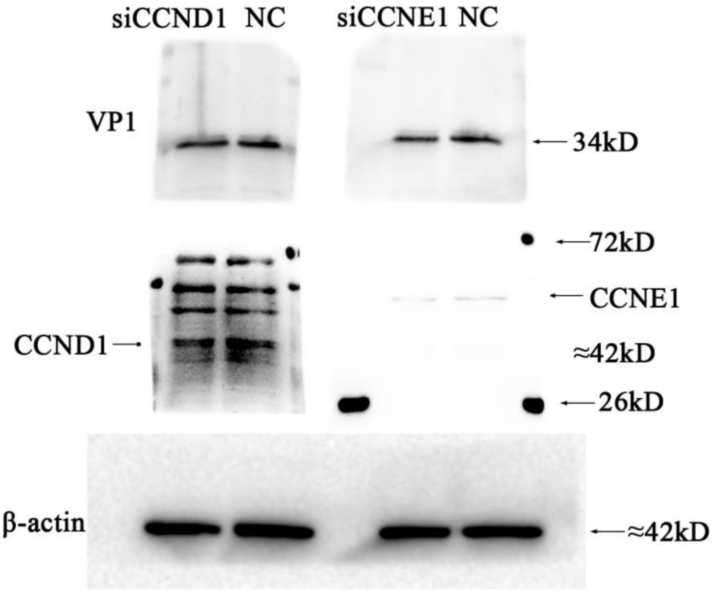

Full unedited gel for figure 6d

The red marks indicate the bands reported in Figure 6d.

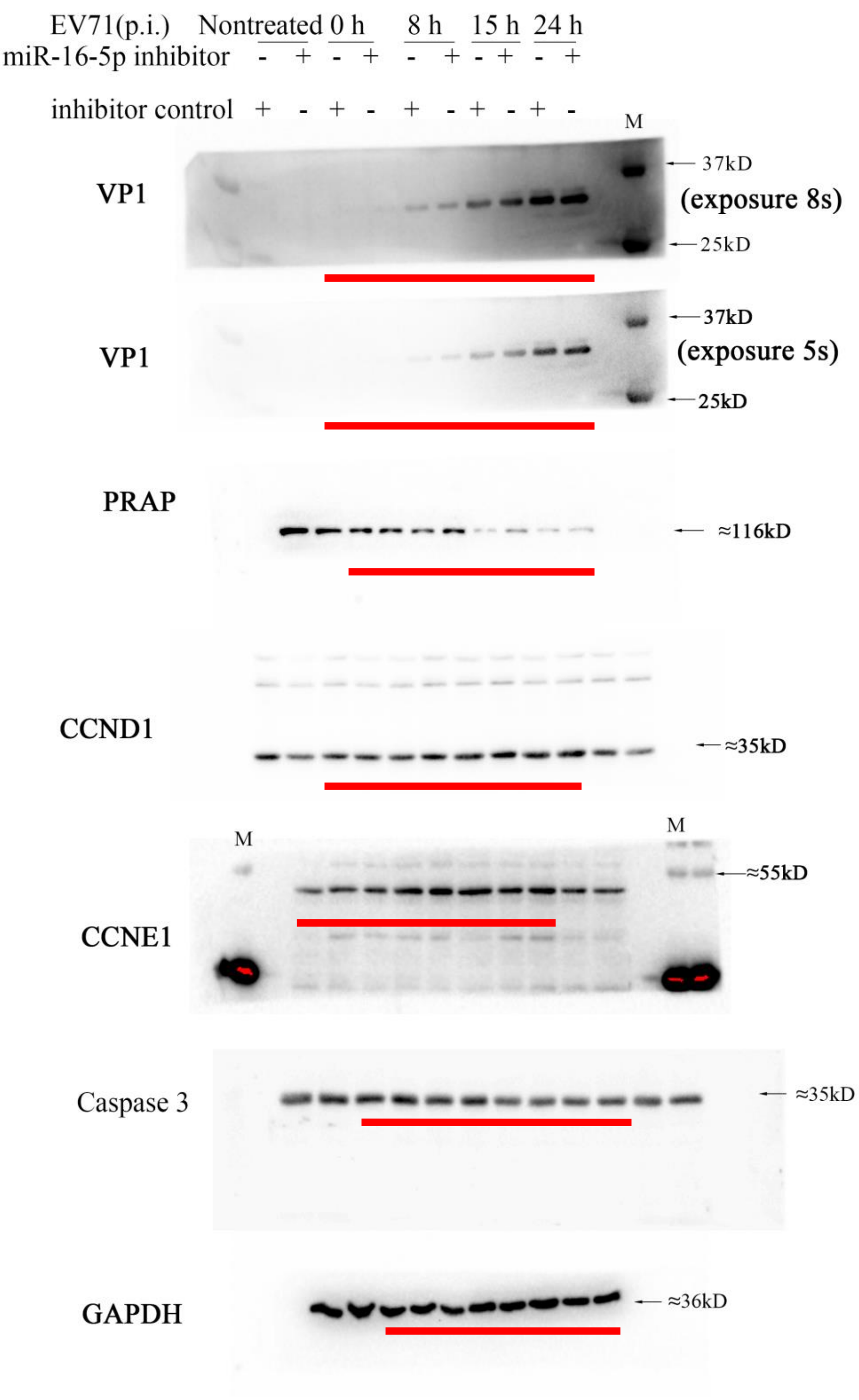

Supplement: Supplementary file 1 — Supplementary Information [file 41598_2017_16616_MOESM1_ESM.pdf]
